# The effect of a game-based mobile app ‘MyHeartMate’ to promote lifestyle change in coronary disease patients: a randomized controlled trial

**DOI:** 10.1093/ehjdh/ztac069

**Published:** 2022-11-24

**Authors:** Robyn Gallagher, Clara K Chow, Helen Parker, Lis Neubeck, David S Celermajer, Julie Redfern, Geoffrey Tofler, Thomas Buckley, Tracy Schumacher, Karice Hyun, Farzaneh Boroumand, Gemma Figtree

**Affiliations:** Susan Wakil School of Nursing and Midwifery, Faculty of Medicine and Health, University of Sydney, Building D17 Johns Hopkins Drive, Sydney, New South Wales 2006, Australia; Charles Perkins Centre, University of Sydney, Building D17 Johns Hopkins Drive, Sydney, New South Wales 2006, Australia; Westmead Applied Research Centre, Faculty of Medicine and Health, University of Sydney, 176 Hawkesbury Road, Westmead, New South Wales 2006, Australia; Department of Cardiology, Westmead Hospital, 176 Hawkesbury Road, Westmead, New South Wales 2145, Australia; Charles Perkins Centre, University of Sydney, Building D17 Johns Hopkins Drive, Sydney, New South Wales 2006, Australia; School of Health Sciences, Faculty of Medicine and Health, University of Sydney, Susan Wakil Health Building, Western Ave, Camperdown, New South Wales 2006, Australia; The Centre for Cardiovascular Health, School of Health and Social Care, Edinburgh Napier University, 9 Sighthill Close, Sighthill, EH11 4QD, UK; Faculty of Medicine and Health, Central Clinical School, University of Sydney, John Hopkins Drive, Camperdown, New South Wales 2006, Australia; Department of Cardiology, Royal Prince Alfred Hospital, John Hopkins Drive, Camperdown, New South Wales 2050, Australia; Clinical Research Group, The Heart Research Institute, 7 Eliza Street, Newtown, New South Wales 2042, Australia; School of Health Sciences, Faculty of Medicine and Health, University of Sydney, Susan Wakil Health Building, Western Ave, Camperdown, New South Wales 2006, Australia; Department of Cardiology, Royal North Shore Hospital, Reserve Road St, Leonards, New South Wales 2065, Australia; Faculty of Medicine and Health, Northern Clinical School, University of Sydney, Reserve Road St, Leonards, New South Wales 2006, Australia; Susan Wakil School of Nursing and Midwifery, Faculty of Medicine and Health, University of Sydney, Building D17 Johns Hopkins Drive, Sydney, New South Wales 2006, Australia; Cardiovascular Discovery Group, Kolling Institute of Medical Research, Reserve Road St, Leonards, New South Wales 2065, Australia; Priority Research Centre for Physical Activity and Nutrition, University of Newcastle, Ring Road, Callaghan, New South Wales 2308, Australia; School of Health Sciences, Faculty of Medicine and Health, University of Sydney, Susan Wakil Health Building, Western Ave, Camperdown, New South Wales 2006, Australia; Susan Wakil School of Nursing and Midwifery, Faculty of Medicine and Health, University of Sydney, Building D17 Johns Hopkins Drive, Sydney, New South Wales 2006, Australia; School of Health Sciences, Faculty of Medicine and Health, University of Sydney, Susan Wakil Health Building, Western Ave, Camperdown, New South Wales 2006, Australia; School of Mathematical and Physical Sciences, Macquarie University, Herring Road, North Ryde, New South Wales 2109, Australia; Department of Cardiology, Royal North Shore Hospital, Reserve Road St, Leonards, New South Wales 2065, Australia; Faculty of Medicine and Health, Northern Clinical School, University of Sydney, Reserve Road St, Leonards, New South Wales 2006, Australia; Cardiovascular Discovery Group, Kolling Institute of Medical Research, Reserve Road St, Leonards, New South Wales 2065, Australia

**Keywords:** Coronary heart disease, Digital health, Risk factors, Randomized controlled trial, Secondary prevention, Mobile health, Gamification

## Abstract

**Aims:**

Secondary prevention reduces coronary heart disease (CHD) progression. Traditional prevention programs including cardiac rehabilitation are under-accessed, which smartphone apps may overcome. To evaluate the effect of a game-based mobile app intervention (MyHeartMate) to improve cardiovascular risk factors and lifestyle behaviours.

**Methods and results:**

Single-blind randomized trial of CHD patients in Sydney, 2017–2021. Intervention group were provided the MyHeartMate app for 6 months. Co-designed features included an avatar of the patient’s heart and tokens earned by risk factor work (tracking, challenges, and quizzes). The control group received usual care. Primary outcome was self-reported physical activity [metabolic equivalents (METs), Global Physical Activity Questionnaire] and secondary outcomes included lipid levels, blood pressure (BP), body mass index, and smoking. Pre-specified sample size was achieved (*n* = 390), age 61.2 ± 11.5 years; 82.5% men and 9.2% current smokers. At 6 months, adjusted for baseline levels, the intervention group achieved more physical activity than control (median difference 329 MET mins/wk), which was not statistically significant (95% CI −37.4, 696; *P* = 0.064). No differences occurred between groups on secondary outcomes except for lower triglyceride levels in the intervention [mean difference −0.3 (95% CI −0.5, −0.1 mmoL/L, *P* = 0.004)]. Acceptability was high: 94.8% of intervention participants engaged by tracking exercise or BP and completing missions; 26.8% continued to engage for ≥30 days. Participants (*n* = 14) reported the app supported tracking behaviours and risk factors, reinforcing and improving self-care confidence, and decreasing anxiety.

**Conclusion:**

A game-based app proved highly acceptable for patients with CHD but did not improve risk factors or lifestyle behaviours other than triglyceride levels.

## Graphical Abstract

**Graphical Abstract:**
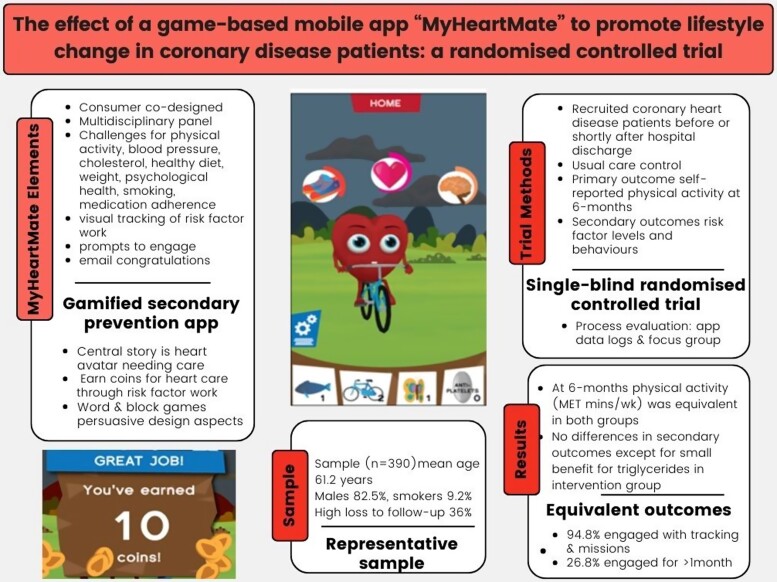

## Background

Despite major advances in the effectiveness of treatments cardiovascular disease (CVD) remains a leading cause of death and health expenditure worldwide.^1^ Reductions in disease progression and major cardiac events can be achieved through secondary prevention strategies, including pharmaceutical and lifestyle behaviours, but uptake of these behaviours is poor and often not sustained.^2,3^ People with CVD continue to smoke (18.5%) and low percentages undertake sufficient physical activity (35.1%) or healthy diet (39.0%) to control risk factors and CVD outcomes.^4^

Cardiac rehabilitation (CR) provides effective support for lifestyle change through programs of exercise and education, and assessing and monitoring risk factors and lifestyle behaviours, but uptake is poor. The efficacy of CR is well-established, with the latest Cochrane Review of 85 trials (*n* = 23 430) reporting reductions in all-cause mortality [risk ratio (RR) 0.87], myocardial infarction (RR 0.72), and hospitalization (RR 0.58).^5^ However, access is far below optimal; only 30% of eligible patients are referred to CR, even less attend and drop-out is common.^6^ Older, female, less educated, uninsured, or unemployed subgroups are the least likely to participate despite having a higher risk of recurrence.^7^ Limited availability of programs create barriers to attendance.^8^ In direct contrast, the widespread availability, uptake and relatively low cost of web-enabled devices such as smartphones and tablets by CVD patients^9^ may overcome access barriers, made evident during COVID-19 pandemic restrictions.^10^ Smartphones and tablet devices can also offer direct access to mobile applications (apps) as a source of secondary prevention support.

Mobile health apps promoting lifestyle change are abundant and recommended for monitoring risk factors and prompting, encouraging and educating regarding lifestyle behaviours in CVD patients.^11^ The most effective and engaging apps incorporate elements of goal-setting, feedback on performance, comparing progress, and competition.^12^ Gamification is an approach that incorporates these features and capitalizes on the innate human desires for competition, social connection, and achievement using rewards, story, and fun.^13^ Inclusion of gamification strategies in a mobile app is theorized to promote both initial and sustained engagement to improve efficacy in treating CVD risk factor outcomes. This potential is indicated in a 2021 systematic review of gamified mobile apps for people with CVD or high CVD risk (seven studies), which reported benefits for HbA1C, physical activity, attitudes to behaviour change, and higher adherence than clinic-based programs.^14^ However, conclusions could not be made due to the poor quality of the evidence. Only two randomized controlled trials (RCT) could be included and no study comprehensively measured risk factors and lifestyle behaviour outcomes.

The aim of this was to evaluate the effect of a game-based mobile app intervention (MyHeartMate) to promote lifestyle change on objective and subjective measures of cardiovascular risk patients with coronary heart disease (CHD).

## Methods

### Study design

**Figure 1:**
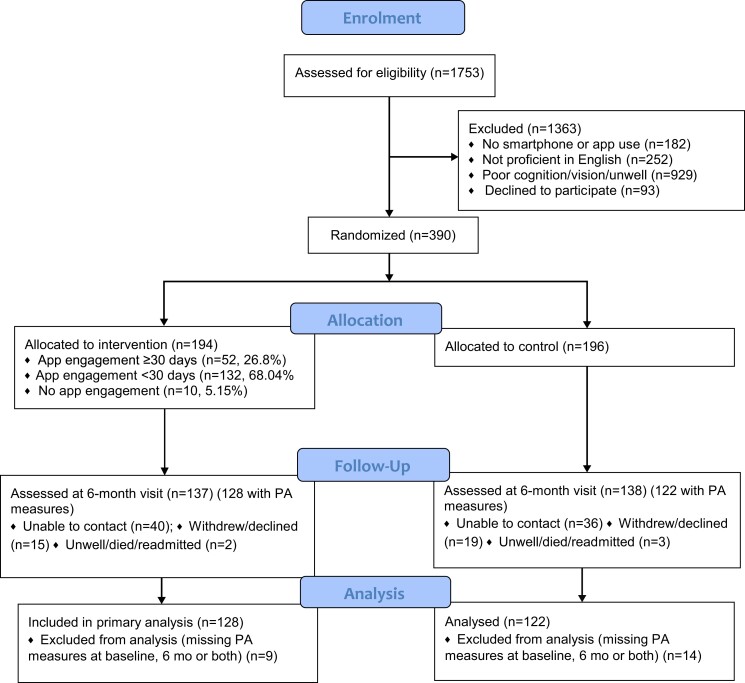
Enrolment of participants in the MyHeartMate randomized clinical trial.

The MyHeartMate study was a parallel-group, two-arm, single-blind, randomized controlled clinical trial that enrolled 390 patients with CHD (*Figure 1*). Treating staff were blinded to assignment as the app is stand-alone, intervention patients were asked not to discuss the app and research staff were independent of treating staff. Objective measures of CVD risk factors [low-density lipoprotein cholesterol (LDL-C), total cholesterol, triglycerides, blood pressure (BP), and anthropometry] including body mass index (BMI, calculated as kg/m^2^) and self-report for physical activity, anxiety, and depressive symptoms were collected at baseline and 6 months post-randomization. Socio-demographic information including marital status, education, and ethnicity were collected by self-report at baseline. Participants were requested to report their own ethnicity, which was later categorized into a fixed set of categories.^15^

The trial was registered with the Australian Clinical Trials Registry (ACTRN12617000869370), and Ethical approval was obtained from all participating hospital sites HREC/17/HAWKE/63. All participants provided written informed consent. The study protocol is published.^16^

### Participants

Patients were recruited during hospitalization for CHD shortly before their discharge or at their first follow-up visit at outpatient clinics (2–3 weeks post-discharge) at three major tertiary referral teaching hospitals and one tertiary referral private hospital in Sydney, Australia. These sites serve Local Health Districts that are densely populated and include areas with some of the highest levels of ethnic diversity and socio-economic disadvantage in Australia. Screening for eligible participants occurred daily through admission and coronary intervention and surgery lists and visits to cardiac outpatient clinics. A log was maintained of all patients screened. The final months of recruitment coincided with the COVID-19 pandemic during which time ward staff screened for potential participants before discharge and advised study personnel, who then called patients by phone.

Patients were eligible if they were adults (≥18 years), had a documented diagnosis of CHD [myocardial infarction (MI), angina, coronary artery bypass graft (CABG), percutaneous coronary intervention (PCI) or 50% or greater stenosis in at least one major epicardial vessel on coronary angiography], were able to provide informed consent, owned a smartphone and were able and willing to use apps. Patients were excluded if they had insufficient English proficiency or a neurocognitive disorder or visual deficit that would limit their capacity to use the app.

### Randomization

Randomization occurred through a computer-generated random number sequence in a uniform 1:1 allocation ratio. An investigator independent of recruitment managed allocation. Recruiting staff enrolled patients by phone call to this investigator and were then advised of the participant’s allocation to group. Control participants received usual care, which included follow-up visits with the cardiac team at 6 weeks, their general practitioner at 1 week and for eligible participants, referral to their local inpatient CR program. Intervention participants in addition to usual care had the app uploaded to their smartphone and were requested to regularly engage with the app for 6 months. The app is open access and intervention participants were encouraged to discuss the app with family but to avoid discussing the app within any patients or staff. All participants received reminders by text message and/or phone call of the 6-month follow-up appointment at 3 and 5 months.

### Intervention

The gamified mobile application (MyHeartMate) secondary prevention intervention was designed to engage participants in CVD lifestyle and risk factor change through tracking behaviours, undertaking short- and longer-term behaviour challenges and related games and quizzes (active behaviour change components). All content was developed by an expert multi-disciplinary panel with reference to the Australian Heart Foundation My Heart My Life program^17^ and was co-designed with consumers through a series of focus-groups working on interface optics, avatar animation, testing tracking, games, and challenges.^18^ Health domains addressed included physical activity, BP, cholesterol, healthy diet, weight, psychological health, smoking, and medication adherence. Development and refinement of the app to maximize engagement and minimize barriers and burden occurred through a co-design process with consumers in a series of focus-groups.

**Figure 2:**
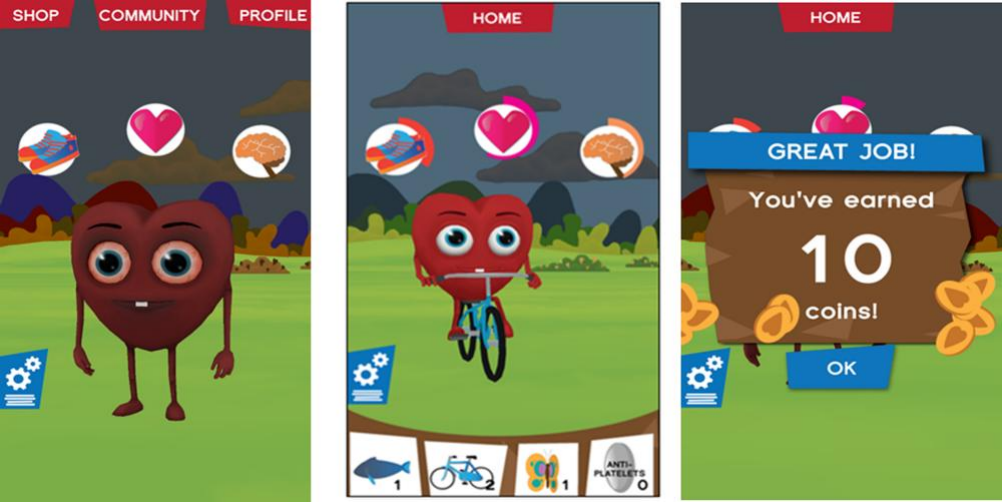
MyHeartMate.

Gamification centred on a cartoon heart avatar as a virtual representation of the user’s health. (*Figure 2*) Users could maintain their own health by completing the active behaviour change components and, by doing so, earn coins to purchase and provide healthy food, exercise, medications, and relaxation items to maintain the health of the heart avatar. Failing to provide daily care to the avatar led to visible decrements to the avatar’s health, activity levels and environment, which compels the user to provide care. Several days absence of care triggered in-app message prompts to provide care. Further game elements incorporated social cognitive theory strategies to provide incremental challenges, ranging from those achievable within-the-day to those requiring behaviour ‘streaks’ of 5 days and 4 weeks to promote sustained behaviour change. Coins earned corresponded with the level of difficulty and congratulatory emails were sent on achievement of substantial behaviour ‘streaks’. Persuasive design elements, harnessing enjoyment, competition, and achievement, encouraged regular app use and included word puzzle and block moving games and visible coin cascade rewards. Personalization was ensured through using participant’s preferred name in messages and greetings, baseline health data (weight, BMI, cholesterol, activity, diabetes, and smoking) as starting points for tracking and social circle via sharing the app and competing on the community leader board.

All intervention-allocated participants received 5 min training on how to upload and log-in to the app and access to a Facebook page providing the same training in video format available on social media. Research staff provided a single call to participants who had not logged-in within 5 days for trouble-shooting advice.

### Procedures

Participants were assessed at baseline and at the outpatient clinic at 6 months as described in the published protocol.^16^ Physical activity was assessed using the Global Physical Activity Questionnaire (GPAQ) with baseline inpatients reporting preadmission physical activity.^19^ Fasting lipids, height, weight, BMI, waist circumference, BP, and heart rate were measured according to international standardized procedures.^16^ Fasting lipids were analysed by local laboratories. Mood was assessed using the Hospital Anxiety and Depression Scale.^20^ Clinical history including medical diagnoses and cardiac medications were collected from the medical record. Current and prior smoking status and socio-demographic data were self-reported. Attendance at CR, emergency department presentations, and hospital admissions were self-reported and cross-checked with medical records. Participants were sent text and email reminders and instructions regarding the follow-up appointment 8, 4, and 1-week/s prior. Four attempts were made to achieve final follow-up including phone call, email, and SMS. Because recruitment sites were referral centres, participants had to travel up to an hour across the city to attend final follow-up. Attempts to reduce travel by planned synchronization with cardiologist appointments was not as successful as anticipated as appointments were highly variable and converted to virtual only during COVID-19 pandemic restrictions. These factors greatly impacted loss to follow-up. During COVID-19 pandemic restrictions research staff were prohibited from direct inpatient staff contact, so study forms were provided to potential participants by ward staff, self-reported baseline physical activity was used instead of the planned use of wearable activity trackers and study assessments were assisted by telephone call or video call by study personnel. These participants also recorded and reported their BMI, waist circumference, BP and heart rate from their home device and from GP-led assessments.

The study primary outcome was total physical activity (MET mins/week) at 6 months as measured by the GPAQ.^20^ Physical activity was selected because of the substantial risk reduction in mortality in this population [adjusted hazards ratio 0.64 (95% CI 0.50, 0.83)].^21^ Physical activity intensity, duration and frequency undertaken at work, for transport and at leisure is assessed by GPAQ, which is interview-administered. The reliability and validity of GPAQ have been reported in a systematic review.^22^

Secondary outcomes included LDL-C, total cholesterol, triglycerides, BMI, waist circumference, BP, heart rate, smoking status, psychological status, attendance at CR, emergency department presentations, and hospital admissions. Given the importance of absolute cardiovascular risk, the combined number of modifiable risk factors above/below target thresholds were calculated for five key guidelines: exercising regularly (≥600 MET mins/wk), LDL-C < 1.8 mmoL/L, BMI <25 kg/m^2^, BP <130/80 mm HG, and not smoking. Outcomes were reported for meeting all five guidelines and the majority (4 of 5) guidelines.^23^

Compliance to intervention was measured by app engagement using data extracted from the app logs (dates of activity, health data tracked and challenges undertaken). App engagement was also measured by self-report survey (frequency, duration, and persistence of app use, device used and sharing with friends and family) for intervention participants. Acceptability, individual likes and dislikes, facilitators and barriers to the usefulness of the app and the effectiveness of different app components were collected using focus groups of three to four participants and led by an experienced facilitator. Focus group participants (*n* = 14) were recruited at follow-up 2019–2020 to ensure representation of genders, study sites, and level of use (</≥30-day use).

### Sample size and data analysis

The a priori sample size estimate was 315, or 390 including an allowance of 20% loss to follow-up. With this sample size and assuming a correlation between baseline and final outcome of *r* = 0.5, there would be 95% power (two-tailed and alpha = 0.05) to detect a difference of 345 (SD 114) MET min/wk between the control and intervention groups.^18^ All analyses were conducted on the basis of intention to treat with participants remaining in their original groups and the statistical analyses followed an a priori plan. Primary and secondary outcome data were locked after data cleaning by the statistician.

Categorical variables have been summarized using frequencies and percentages, and numeric variables are summarized using mean and standard deviation for normally distributed data and median and interquartile interval for skewed data. Participants were classified as compliant/engaged with the app based on whether their data-log record indicated they had tracked exercise and/or BP ≥5 times and completed ≥18 missions of any type and the last date of app use was equal or more than 30 days from their first log-in (study enrolment). The 30-day period of engagement was selected from a systematic review of 52 mobile apps that identified the effectiveness of therapeutic persuasiveness on outcomes at this time.^24^

The primary outcome was analysed using robust multivariable regression as the primary outcome had a considerable number of outliers with a skewed distribution. The model was adjusted for the baseline value of the primary outcome. After checking the range and the distribution of the continuous secondary outcomes, analysis of covariance was used to compare groups at 6 months with baseline outcome values as a covariate. For binary secondary outcomes, including meeting combined CVD risk guidelines, relative risk was calculated and compared between groups at 6 months using log-binomial regression, adjusting for the baseline outcome measure.

As more than 5% of the primary outcome data were missing at follow-up, baseline characteristics of patients with missing and not missing primary outcome data were compared. The data were missing at random or possibly missing not at random as patients with missing primary outcome data at follow-up had lower median GPAQ METs at baseline. Sensitivity analyses using complete case analysis and multiple imputation was conducted. For multiple imputation, auxiliary variables used to estimate missing data were: GPAQ METs at follow-up, age, gender, education, employment, diagnosis, meeting 4/5 guidelines, depression score, anxiety score, randomized groups, and GPAQ METs at baseline. All statistical tests were two-tailed with the significance level set at 0.05. All analyses were conducted using SAS version 9.4 (SAS Institute Inc.).

## Results

From January 2017 to January 2021, 1750 patients were screened, 390 were enrolled and randomized (1270 did not meet the eligibility criteria and 93 declined participation) (*Figure 1*). Recruitment closed when the study sample size was reached. At final follow-up, 76 were unable to be contacted, 34 withdrew/declined, 5 had died or were too unwell, and physical activity data (METs) were available for 250 (64.1% of randomized patients; 63.4% of those allocated to the intervention group, and 62.8% of those allocated to the control group). Participants who completed follow-up were more likely to be university graduates, married, overweight/obese and had more depressive symptoms and were less likely to have STEMI diagnosis. The median time to follow-up was 6.9 months. The whole sample at baseline had a mean age of 61.2 years, 82.5% were men, means were for LDL-cholesterol 2.4 mmoL/L, BP 121.3/72.6 mm Hg and BMI 28.4 km/m^2^ and 9.2% were current smokers (*Table 1*). Baseline MET mins/wk were a median of 1800 (IQI 920–2900). There were no statistically significant differences between intervention and control groups on any characteristic at baseline.

**Table 1:** Baseline characteristics

Variable	Total *n* (%) *n* = 390	Intervention *n* (%) *n* = 194	Control *n* (%) *n* = 196	*P* level
Age, mean (SD)	61.2 (11.5)	60.9 (11.9)	61.5 (11.2)	0.61
Male	320 (82.5)	158 (81.4)	162 (83.5)	0.59
≥High school education	338 (86.7)	166 (85.6)	172 (88.6)	0.52
Married/partnered	302 (77.8)	151 (77.8)	151 (77.8)	0.91
Ethnicity				
ȃOceanian (incl Australian)	202 (52.2)	96 (49.5)	106 (54.9)	0.32
ȃNorth-West European	53 (13.7)	31 (16)	22 (11.4)	
ȃSouthern/Central Asian	42 (10.9)	26 (13.4)	16 (8.3)	
ȃSouthern/Eastern European	21 (5.4)	12 (6.2)	9 (4.7)	
ȃSouth-East Asian	21 (5.4)	10 (5.2)	11 (5.7)	
ȃOther	36 (9.2)	19 (9.8)	17 (8.8)	
Employed (full/part/casual)	240 (61.9)	117 (60.3)	123 (63.4)	0.78
**Clinical data**				
Diagnosis^a^				
ȃSTEMI +/− PCI	115 (30)	59 (31.1)	56 (29)	0.84
ȃNon-STEMI +/− PCI	93 (24.3)	47 (24.7)	46 (23.8)	
ȃAngina +/− PCI	175 (45.7)	84 (44.2)	91 (47.2)	
ȃCABG	104 (26.7)	56 (28.9)	48 (24.5)	
**Risk factors**				
ȃBMI kg/m^2^, mean (SD)	28.4 (5.4)	28.2 (5.8)	28.5 (4.9)	0.64
ȃBMI > 25 kg/m^2^	166 (42.6)	84 (43.3)	82 (41.8)	0.27
ȃWaist circumference cm, mean (SD)	99.9 (14.7)	99.7 (16)	100.2 (13.2)	0.74
Cholesterol mmoL/L				
ȃTotal cholesterol mmoL/L, mean (SD)	4.1 (1.3)	4.2 (1.3)	4 (1.2)	0.25
ȃLDL-C mmoL/L mean (SD)	2.3 (1.1)	2.4 (1.1)	2.2 (1)	0.19
ȃTriglycerides mmoL/L, mean (SD)	1.6 (0.9)	1.6 (1)	1.6 (0.9)	0.85
Blood pressure, mm Hg				
ȃSystolic, mean (SD)	120.9 (16.6)	119.8 (16.7)	121.9 (16.5)	0.16
ȃDiastolic, mean (SD)	72.6 (10.3)	72.2 (10.7)	73.1 (10)	0.38
Heart rate, b/min	71.4 (11.6)	71.5 (12.1)	71.3 (11)	0.96
Current smoking^b^	37 (9.5)	18 (9.3)	19 (9.7)	0.88
Diabetes	92 (23.6)	41 (21.1)	51 (26)	0.25
Hypertension	184 (47.2)	88 (45.4)	96 (49)	0.47
Total physical activity (MET min/wk) median (IQI)	1800 (920, 3900)	1720 (900, 3720)	1960 (960, 4200)	0.21
Inactive: (<600 MET min/wk)^b^	54 (16.3)	26 (15.4%)	28 (17.2%)	0.65
Mood state				
Anxiety, mean (SD)	4.9 (4)	5.1 (4.1)	4.8 (3.9)	0.41
Depression, mean (SD)	3.4 (3.1)	3.5 (3.2)	3.2 (3.0)	0.24
**Achieving guideline levels^b^**				
Exercising regularly (5 days of 30 mins/day)	278 (83.7)	143 (84.6)	135 (82.8)	0.66
LDL-C < 1.8 mmoL/L	111 (35.7)	51 (32.7)	60 (38.7)	0.26
BMI <25 kg/m^2^	95 (24.8)	56 (29.2)	39 (20.4)	0.05
Blood pressure <130/80 mm Hg	217 (56.4)	115 (59.9)	102 (52.8)	0.16
Non-smoker	353 (90.5)	176 (90.7)	177 (90.3)	0.88
Meeting 5 guidelines	14 (3.6)	8 (4.1)	6 (3.1)	0.57
Meeting 4/5 guidelines	99 (25.4)	53 (27.3)	46 (23.5)	0.38
**Medications**				
ACE inhibitor/ARB	116 (29.7)	35 (18.0)	39 (19.9)	0.14
Aspirin	341 (87.4)	166 (85.6)	175 (89.3)	0.26
Β-Blocker	237 (60.8)	120 (61.9)	117 (59.7)	0.66
Statin	332 (85.1)	161 (83.0)	171 (87.2)	0.24
Anticoagulant	85 (21.8)	40 (20.6)	45 (23.0)	0.57
P2Y12	203 (52.1)	100 (51.5)	103 (52.6)	0.84

STEMI, ST-elevation myocardial infarction; PCI, percutaneous coronary intervention; CABG, coronary artery bypass graft surgery; LDL-C, low-density lipoprotein cholesterol; BMI, body mass index; MET, metabolic equivalents.

Multiple diagnoses possible.

Relative risk (95% confidence interval).

### Effect on primary and secondary outcomes

At 6 months outcome after adjusting for baseline MET mins/wk, the intervention group (*n* = 128) was achieving more physical activity in MET mins/wk than the control group (*n* = 122) at a median difference of 329; however, this was not statistically significant (95% CI −37.4, 696; *P* = 0.064) (*Table 2*). The intervention group (*n* = 91) also had lower triglyceride levels than the control group (*n* = 80) at mean difference of −0.3 (95% CI −0.5, −0.1 mmoL/L, *P* = 0.004) when adjusted for baseline levels. However, there were no differences between the groups on any other secondary outcome measured.

**Table 2:** Primary and secondary outcomes at 6 months

Parameter	InterventionMedian (IQI)		ControlMedian (IQI)		Mean difference or relative risk (95% CI)		*P*-value for difference
Primary outcome^a^							
Total physical activity (MET min/wk)	1860	(840, 3600)	1570	(800, 3000)	1	(−37, 696)	0.064
Secondary outcomes^a^							
	Mean (SD)		Mean (SD)				
ȃBMI kg/m^2^	27.6	(5.6)	28.0	(4.7)	−0.5	(−1.1, 0.2)	0.18
ȃWaist circumference cm	98.4	(14.6)	100.2	(13)	−1.8	(−4.2, 0.6)	0.14
Cholesterol							
ȃTotal cholesterol (*n* = 171)	3.5	(0.8)	3.7	(0.8)	−0.1	(−0.4, 0.1)	0.33
ȃLDL-C (*n* = 166)	1.8	(0.6)	1.8	(0.7)	0	(−0.3, 0.2)	0.72
ȃTriglycerides (*n* = 171)	1.2	(0.6)	1.5	(0.9)	−0.3	(−0.5, −0.1)	0.004
Blood pressure, mm Hg							
ȃSystolic	107.2	(13.7)	108.9	(14.6)	−1.7	(−6.3, 2.9)	0.47
ȃDiastolic	73.6	(10.1)	73.9	(9.6)	−0.3	(−2.5, 1.8)	0.76
Heart rate b/min	59.5	(12)	61.1	(10.7)	−1.6	(−4, 0.8)	0.20
	*n*	(%)	*n*	(%)			
Current smoker	4	(3)	7	(5.1)	0.59	(0.2, 1.9)	0.39
Inactive: (<600 MET min/wk)	15	(11.8)	21	(17.2)	0.91	0.5, 1.7)	0.76
Mood state^a^							
Anxiety	4.2	(3.8)	4.0	(3.4)	0.2	(−0.5, 0.8)	0.65
Depression	2.8	(3.2)	2.7	(2.7)	0.1	(−0.5, 0.7)	0.77
Achieving guideline levels^c^							
Exercising regularly (5 days/wk for 30 mins)	112	(88.2)	101	(82.8)	1	(0.91, 1.09)	0.23
LDL-C < 1.8 mmoL/L	45	(49.5)	42	(56)	1.07	(0.77, 1.47)	0.69
BMI <25 kg/m^2^	46	(37.7)	27	(21.6)	1.32	(0.98, 1.78)	0.067
BP <130/80 mm Hg	63	(52.1)	60	(48)	0.94	(0.77, 1.16)	0.59
Non-smoker	180	(93.8)	190	(97.9)	0.99	(0.96, 1.01)	0.33
Meeting 5 guidelines	9	(4.7)	4	(2.1)	2.16	(0.68, 6.89)	0.19
Meeting 4/5 guidelines	41	(21.4)	30	(15.5)	1.17	(0.79, 1.74)	0.43
Other outcomes^c^							
ED presentations, *n* (%)	24	(17.6)	18	(13.1)	1.34	(0.76, 2.36)	0.30
Hospital admissions, *n* (%)	23	(16.8)	25	(18.2)	0.92	(0.55, 1.54)	0.75
Cardiac rehab attendance,	99	(72.3)	85	(62.0)	1.16	(0.99, 1.38)	0.07

Analysis of covariance including randomized groups (intervention vs. control) and adjusted for baseline value for continuous measures; MET, metabolic equivalents.

β estimate (95% CI).

Relative risk (95% confidence interval).

### Effect on achieving combined risk factor behaviours

Participant’s control of individual modifiable risk factors at 6 months was suboptimal except for exercising regularly (88.2 and 82.8%) and being a non-smoker (93.8 and 97.9%). There was no difference between groups for achieving guideline levels for exercising regularly, LDL cholesterol, BMI <25 kg/m^2^, BP <130/80 mm Hg or being a non-smoker, or for when these risk factors were combined (*Table 2*). Achievement of guidelines at 6 months occurred in LDL-C for 31.7%, BP (32.2%), regular exercise (9.5%), BMI (9.4%), and smoking (6.9%) with small proportions no longer achieving guidelines (1–8%) and no differences between groups (see Supplementary material online, *Table S1*).

### Other outcomes

During the 6-month follow-up, 42 (15.4%) presented to the ED, 48 (17.6%) were admitted to hospital and 184 (67.6%) attended CR. There were no differences between groups on these outcomes.

### Process measures

Of the 1363 patients hospitalized with CHD screened for eligibility the main reasons for exclusion were poor cognition or vision, being too unwell or not proficient in English (1181, 86.6%) or did not own a smartphone, or if they did, had never used an app (182, 13.3%).

Intervention participants (*n* = 194) were classified as compliant with the intervention if they were ‘engaged with the app’ through tracking exercise and/or BP ≥5 times and completing ≥18 missions of any type (data extracted from app logs). On this basis, 94.8% were engaged: 26.8% for ≥30 days and 68.0% for <30 days. Self-reported (*n* = 124) app interaction occurred most often at ≥2/week (35.3%) and ≥5 min/session; 16.3% still used the app at 6 months and 45.5% had shown the app to family/friends (*Table 3*).

**Table 3:** Self-reported app engagement (*n* = 124)

Parameter	*n*	%
Frequency of usage		
ȃ≥twice/day	9	(7.4)
ȃ1/day	15	(12.3)
ȃ2–5/week	19	(15.6)
ȃ ≤ 1/week	79	(64.8)
Duration of usage/session		
ȃ15–30 min	9	(7.4)
ȃ5–15 min	32	(26.4)
ȃ < 5 min	80	(66.1)
ȃ6 months		
Currently using	20	(16.3)
ȃ1/day	18	(14.5)
ȃ ≥ 2/day	2	(1.6)
Showed to family/friends	56	(45.5)

Participant (*n* = 14) interviews revealed that the app was considered helpful to track behaviours and risk factors, prompting to seek information, improving confidence in self-care, decreasing anxiety, and reinforcing what participants needed to do for their heart health, especially in the early discharge period. However, participants stopped using the app over time when the perceived return on the effort of app engagement decreased or they found other apps that suited their purpose better. Some participants stopped engaging very early because they had difficulties understanding the app functions and/or purpose or they didn’t like the overall game concept.

## Discussion

This trial is one of the first to evaluate the effect of a stand-alone gamified mobile app designed to promote secondary prevention lifestyle behaviours in patients being discharged from hospital following a CHD event. Our current well-designed and high quality RCT contributes to the field by demonstrating in an acute CHD population that a mobile app with game features results in relatively neutral behaviour change effects at 6 months. Compared with usual care, use of the MyHeartMate app did not have statistically significant effects on CHD risk factors or secondary prevention behaviours. While promise was evident for improved physical activity and CR attendance only triglyceride levels were significantly reduced. The need for risk factor modification support was evident as multiple areas of risk were not at guideline level. MyHeartMate proved feasible and acceptable despite the stand-alone and unique gamified nature of the app, for a very ethnically diverse and socio-economically disadvantaged population and in providing remote support during the COVID-19 pandemic. The relative lack of effect could potentially be that the initial early uptake and active engagement supported by game features was not sustained beyond 1 month in the majority and was most likely insufficient to impact the outcomes measured.

The study tested the concept of gamification to promote engagement and deliver behaviour change principles. The study app is the first stand-alone app for CVD to incorporate game strategies and to use a virtual heart as the ‘story’ for the behaviour change components. Effectiveness of such behaviour change apps depend on the propensity to use the apps and the level of engagement.^25,26^ Stand-alone apps for CVD (hypertension), which include strategies within the gamification umbrella have reported limited benefits in a systematic review of eight studies (*n* = 1657).^27^ The review indicated a small improvement in systolic BP but no benefit for physical activity. App engagement was highlighted as an important influence^26,28^ and may affect the degree of behavioural activation^14^ at least for weight and BMI, yet few studies objectively measure engagement^12,14^ or rigorously test apps in the older CHD population.

App engagement is indeed complex, difficult to forecast accurately and may be independent of the number and type of features incorporated and more dependent on user perceptions.^13,14^ We used an iterative consumer co-design process to develop the app, minimize app burden and glitches and maximize appeal of the heart avatar and game components.^9,16^ We also used data logs to objectively measure engagement with multiple app components and over time. We found that engagement with the behaviour change components (classified as having tracked exercise and/or BP ≥5 times and completed ≥18 missions) was almost universal at 94.8% but was not sustained. Only 26.8% engaged for at least 30 days, the duration considered necessary for effective activation of lifestyle behaviours.^13^ App engagement may be dependent on user needs at the time in recovery from ACS.^18^ We recruited patients being discharged or shortly after discharge from hospital, a stressful and anxious time, especially during the COVID-19 pandemic. It is possible that low burden, more passive, phone-based interventions, such as one-way personalized text messaging, may be more suitable at this time and certainly these methods have been able to achieve comprehensive behaviour change.^29,30^ While the consumers used in the co-design process included a patient early in ACS recovery, most of the consumers were CR graduates. Future app development should include consumers with more recent experience. More in-depth understanding is needed on the relationship between engagement with specific app components, which distinguishes game features, and behaviour change outcomes and in different populations and contexts such as pandemics. Conventional study designs such as RCTs need to be reconsidered so the relative effect of different app components relate to uptake vs. ongoing engagement in CHD populations.^31^

Limited benefits of the app were evident. Patients using the MyHeartMate app lowered their triglyceride levels by 25% at 6 months, 19% more than the usual care group. This reduction in triglycerides was achieved independent of clinician time and was larger in magnitude than that achieved by high intensity dietary counselling (≥6 × 30 min sessions) (−0.31 vs. −0.24 mmoL/L) in a systematic review of 22 trials in a similar-aged cardiometabolic disease population.^32^ High triglycerides, especially combined with low high-density lipoprotein (HDL) cholesterol (ratio), are predictors of mortality and myocardial damage independent of LDL-C and lipid treatments in CHD patients.^33^ App users also improved their physical activity participation by median 140 MET mins/wk, whereas the usual care group had decreased physical activity by median 150 MET mins/wk in 6 months. These differences were not statistically significant (*P* = 0.078), possibly due to the wide variation in physical activity being reported and dilution of effects from an unusually high CR participation rate of 67.6% vs. typical rates of 35–50%.^7,8^ Regardless, physical activity participation provides substantial benefits to patients with CHD including decreasing the progression of plaques and improving recruitment of collaterals, endothelial function and coronary blood flow.^34^ Guidelines recommend at least 150 min/week of moderate exercise, so an improvement of 140 MET mins/wk in the MyHeartMate group is likely to have a clinical impact.^35^ Future research is needed that can distinguish the most active and effective app components including gamification elements for specific behaviours in this population.

Several limitations apply to this study that need to be considered. Loss to follow-up was higher than planned and missing data occurred for the primary outcome so the follow-up sample size for analyses of 315 was not achieved, increasing the possibility of Type 2 error. Loss to follow-up was equal in the intervention and control groups so app-related issues including app burden or dislike were unlikely causes. Other potential explanations include the burden of travel and time to attend the follow-up clinic and the pivot in study methods to phone, email, and mail enforced by COVID-19 pandemic restrictions as well as related impact on patients and staff for participants recruited later in the study.

Participants who completed follow-up differed to those who did not in several areas, all of which were included as auxiliary variables to impute the primary outcome and sensitivity analyses conducted. The primary outcome of objective physical activity recorded by activity trackers was planned, but a pivot to self-report was required due to COVID-19 restrictions to patients. Self-reported physical activity may be affected by difficulties in recall and estimation of physical activity. While significant effects of the intervention were demonstrated on triglyceride levels, effects need to occur on multiple risk factors and the sample size appropriate to affect clinical outcomes. Recruitment was deliberately inclusive of populations with high levels of ethnic diversity and socio-economic disadvantage. However, the sample achieved had high levels of education (87% ≥high school) and BP control (mean 121/73 mmHg) perhaps indicating that research participation or the app itself lacked appeal. Future research should carefully track reasons for study refusal. The study was conducted in one city so further investigation is required in non-metropolitan areas and in multiple national and international contexts and following translation of the app content to other languages. Finally, it was impossible to completely blind the trial as participants knew whether they were allocated to the app intervention. However, recruiting staff were blind to allocation at the time of recruitment and participants were asked not to disclose their group allocation to treating staff or other patients. A standard approach to assessment of acceptability such as the System Usability Scale would potentially provide insight into drop-out and decline in engagement and future research should incorporate these methods and a larger sample for the user focus groups.

The use of a novel game-based mobile app designed to promote behaviour change and risk factor reduction did not prove effective in improving risk factors or lifestyle behaviour change in patients with CHD. Potential benefits for triglyceride levels and physical activity occurred. High levels of acceptability were evident but engagement was not sustained by many for the time required for effectiveness. Future research is needed that can distinguish the most active and effective app components for behaviour change in this population.

## Supplementary Material

ztac069_Supplementary_Data

## Data Availability

The data collected for this study is not available to the public due to human research ethics restrictions.
