# Supplementary material for: The effect of a game-based mobile app ‘MyHeartMate’ to promote lifestyle change in coronary disease patients: a randomized controlled trial
Source: Eur Heart J Digit Health. 2022 Nov 24;4(1):33–42. doi: 10.1093/ehjdh/ztac069 (PMC9890079; doi:10.1093/ehjdh/ztac069)
Supplement: ztac069_Supplementary_Data [file ztac069_supplementary_data.docx]

Supplementary Table 1 Change in achievement of cardiovascular disease risk factor guidelines from to 6-months compared by group

| Guideline | Baseline | | | | 6-months | | | | | | | | | | | |
| --- | --- | --- | --- | --- | --- | --- | --- | --- | --- | --- | --- | --- | --- | --- | --- | --- |
|  | Achieved | | | | Switch to achieved* | | | | Switch to not achieved | | | | No change | | | |
|  | Intervention | | Control | | Intervention | | Control | | Intervention | | Control | | Intervention | | Control | |
|  | n | % | n | % | n | % | n | % | n | % | n | % | n | % | n | % |
| Exercising regularly (5 days @ 30 mins/day) | 143 | 84.6 | 135 | 82.8 | 14 | 11.8 | 8 | 7.2 | 8 | 6.7 | 8 | 7.2 | 97 | 81.2 | 95 | 85.6 |
| LDL-C <1.8 mmol/L | 51 | 32.7 | 60 | 38.7 | 22 | 2.3 | 18 | 31.0 | 4 | 5.9 | 8 | 13.8 | 42 | 61.8 | 32 | 55.2 |
| BMI <25 kg/m^2^ | 56 | 29.2 | 39 | 20.4 | 13 | 10.7 | 10 | 8.2 | 6 | 4.9 | 9 | 7.4 | 103 | 84.4 | 103 | 84.4 |
| Blood pressure <130/80 mm Hg | 115 | 59.9 | 102 | 52.8 | 40 | 33.1 | 39 | 30.9 | 3 | 2.4 | 1 | 0.8 | 78 | 64.4 | 86 | 68.3 |
| Nonsmoker | 176 | 90.7 | 177 | 90.3 | 10 | 5.5 | 17 | 8.7 | 4 | 2.1 | 2 | 1.0 | 79 | 92.7 | 175 | 90.2 |

*Sample size reduced at 6-months to those who had data at baseline and 6-months: for exercise (n=230), LDL-C (n=126), BMI (n=244), Blood pressure (n= 245)
